# Supplementary material for: Parallax attention stereo matching network based on the improved group-wise correlation stereo network
Source: PLoS One. 2022 Feb 9;17(2):e0263735. doi: 10.1371/journal.pone.0263735 (PMC8827418; doi:10.1371/journal.pone.0263735)
Supplement: Attachment — Submitted filename: Response to Reviewers.docx [file pone.0263735.s001.docx]

Dear Editors and Reviewers:

Thank you for giving us the opportunity to submit a revised draft of the manuscript “Parallax attention stereo matching network based on the improved group-wise correlation stereo network” for publication in the Journal of “***PLOS ONE***”. We appreciate the time and effort that you and the reviewers dedicated to providing feedback on our manuscript and are grateful for the insightful comments and valuable improvements to our paper. We have studied comments carefully and have made correction which we hope meet with approval. Revised portion are marked in red in the paper. The main corrections in the paper and the responds to the reviewer’s comments are as flowing:

Responds to the reviewer’s comments:

Reviewer #1:

Major Concerns:

1) Parallax attention has been widely used for various of stereo image processing tasks, such as stereo image super-resolution [c1-c3], stereo matching [c4], stereo image dehazing [c5], and stereo color transfer [c6]. The authors are suggested to provide a comprehensive review of the recent progress of parallax attention in the related work section.

[c1] Symmetric parallax attention for stereo image super-resolution, CVPRW 2021.
[c2] A stereo attention module for stereo image super-resolution, SPL 2020.
[c3] Feedback Network for Mutually Boosted Stereo Image Super-Resolution and Disparity Estimation, arxiv 2021.
[c4] Parallax Attention for Unsupervised stereo correspondence learning, TPAMI 2020.
[c5] BidNet: Binocular image dehazing without explicit disparity estimation, CVPR 2020.
[c6] Asymmetric stereo color transfer, ICME 2021.

**The authors’ Answer:** We are very grateful to your comments for the manuscript, your suggestion is of great help to our work. According to your advice, we amended the relevant part in manuscript. We have taken the following measures to this advice: Firstly, we have changed the expression of “Despite the excellent performance of the attention module, it rarely appears in stereo matching network” in the beginning of our previous abstract to “Despite the excellent performance of the attention module, the parallax information provided by binocular images has not been fully utilized.” Secondly, we have made a comprehensive review of the recent progress of parallax attention in the introduction and related work section. We introduced the progress of recent works such as PASMnet, PASSRnet, SISRnet, etc. For more details, please check the latest manuscript. Thanks again for your advices.

2) Since parallax attention has already been used for stereo matching task in [c4], the main difference between the proposed network and PASMnet [c4] should be well addressed and discussed.

**The authors’ Answer:** We are extremely grateful to you for pointing out this problem. To show the difference between our proposed PA-Net and PASMnet, we have discussed in the introduction and related work section that although PASSR-Net and PASMnet have made some progress in stereo image super-resolution, their main concern is the parallax information on two-dimensional (height and width) level, that contributes little compared with garnering attention to parallax information directly in three-dimensional (disparity, height and width) level. As the supporting data of our conclusion, we have made a comparison between our proposed PA-Net and PASMnet in Table 4. which shows that PA-Net performance better than PASMnet on KITTI 2015 dataset. For more details, please check the latest manuscript. Thanks again for your advices.

Minor Concerns:

1) In table 1, the resolution of the 2nd to 4th branches of the PA module is 1/4 x 1 x 1/4H x 1/4 W x 32(or64). The first 1/4 is confusing. Is it the batch dimension?

**The authors’ Answer:** We are sorry to tell you that this is the wrong expression due to our mistakes, the exact expression is 1 x 1/4 H x 1/4 W x 32(or64). 1 represents the value of the parallax dimension, and we have revised the correct result in the manuscript file.

Special thanks to you for your good comments.

Reviewer #2

Minor Concerns:

1) It should be noted that parallax attention mechanism has already been used for disparity estimation in PASMnet [r1]. However, the method proposed in this paper is significantly different from that in [r1]. The authors are strongly suggested to discuss the difference between their method and PASMnet.

**The authors’ Answer:** We are extremely grateful to you for pointing out this problem. We have discussed the difference between our proposed PA-Net and PASMnet. As the supporting data of our conclusion, we have made a comparison between PA-Net and PASMnet in Table 4. which shows that PA-Net performance better than PASMnet on KITTI 2015 dataset. For more details, please check the latest manuscript. Thanks again for your advices.

2) Since this paper focuses on the parallax attention, some recent advances in parallax attention should be reviewed in the related work (e.g., symmetric parallax attention [r2], cross parallax attention [r3], stereo attention [r4] and so on) to make the survey of the literature more comprehensive.

**The authors’ Answer:** We are very grateful to your comments for the manuscript, and we have made a comprehensive review of the recent progress of parallax attention in the introduction and related work section. We introduced the progress of recent works such as PASMnet, PASSRnet, SISRnet, etc. For more details, please check the latest manuscript. Thanks again for your advices.

3) The layout of the tables and figures in this paper need to be further improved. A table cannot be placed on two separate pages.

**The authors’ Answer:** Thanks for your advice, we have revised this issue in the latest manuscript and we have also made some changes to table 1. For more details, please check the latest manuscript. Thanks again for your advices.

Special thanks to you for your good comments.

We tried our best to improve the manuscript and made some changes in the manuscript.  These changes will not influence the content and framework of the paper. We appreciate for Editors/Reviewers’ warm work earnestly, and hope that the correction will meet with approval.

Once again, thank you very much for your comments and suggestions。

Yours sincerely,

Jinan Gu
