# Supplementary material for: Parallax attention stereo matching network based on the improved group-wise correlation stereo network
Source: PLoS One. 2022 Feb 9;17(2):e0263735. doi: 10.1371/journal.pone.0263735 (PMC8827418; doi:10.1371/journal.pone.0263735)
Supplement: Attachment — Submitted filename: Response to Reviewers.docx [file pone.0263735.s002.docx]

Dear Editors and Reviewers:

Thank you for giving us the opportunity to submit a revised draft of the manuscript “Parallax attention stereo matching network based on the improved group-wise correlation stereo network” for publication in the Journal of “***PLOS ONE***”. We appreciate the time and effort that you and the reviewers dedicated to providing feedback on our manuscript and are grateful for the insightful comments and valuable improvements to our paper. We have studied comments carefully and have made correction which we hope meet with approval. Revised portion are marked in red in the paper. The main corrections in the paper and the responds to the reviewer’s comments are as flowing:

Responds to the reviewer’s comments:

Reviewer #3:

This paper presents a new network for the stereo correspondence problem. From the comparison, it does look like the proposed framework outperforms other works. The structure of the network is clearly explained. Generally, the work looks good. However, the writing of the paper is far from ready.

First of all, the paper jump into the specific technique and problem directly from the beginning. There is not much background introduction and concept explanation for normal readers. If the reader (even doing computer vision research) are not familiar with this specific research, it will be very difficult to understand the work and follow the writing. I do suggest the author to use the introduction section to clearly explain the problem, instead of review some papers.

**The authors’ Answer:** We are very grateful to your comments for the manuscript, your suggestion is of great help to our work. According to your advice, the logical relationship is reorganized and the introduction part was completely modified.

Secondly, this paper made the same mistake as lots of machine learning papers. It only explains the proposed network structure then jump directly into the experiment, using the result to show the effectiveness of the new network. There is no clear explanation why this network is designed in this specific way. It is pretty much like, I try this and “luckily” the result is good. For a good academic writing, there should be a clear motivation and discussion on the design. All the decision should be clearly justified.

**The authors’ Answer:** We are extremely grateful to you for pointing out this problem. Based on your advice, in the method part, we made a clear explanation of the reasons for the model design.

1. In the abstract, the paper targets at “ill-posed areas (weak texture, repeat texture and occlusion regions)”. However, in the paper, all those problems are not mentioned specifically in the experiment.

**The authors’ Answer:** We are extremely grateful to you for pointing out this problem. We have made the latest changes in the abstract part.

2. In the abstract, how the “novel edge detection branch and multi-scale cost volume” will “obtain finer texture features”. Is there a justification for this claim or “common sense”?

**The authors’ Answer:** We use the edge loss function to learn the edge cues of the images. It is demonstrated that based on our model, edge detection project is conducive to improve the accuracy of disparity estimation.

3. The English needs to be polished. For example, I am not sure the following expression are correct or not, please check: “word goal characteristic”, “aggressive universal overall performance”, “conference models”, “competitive advantage”. And there are lots of long sentences which should be broken into separate ones (for example line 124-127). And in line 217, “Which” should be merged into previous sentence. Line 309, “Table 5, demonstrate”, no comma. There are lots of small mistakes.

**The authors’ Answer:** We are very grateful to your comments for the manuscript, we have revised your question accordingly, for details, you can view the latest manuscript.

4. Line 77, “Our PA-Net outperformed the state-of-the-art GWC-Net [11] in ill-posed regions”, this should be put into the contribution.

**The authors’ Answer:** We are very grateful to your comments for the manuscript, we have revised the manuscript based on your advice.

5. Line 45-54 list quite a few papers, however, the logic behind this discussion is not clear. How these literature review helps the overall argument.

**The authors’ Answer:** We are very grateful to your comments for the manuscript, we have revised the manuscript based on your advice.

6. Line 135-136 should have an overall introduction about the whole methodology.

**The authors’ Answer:** We are extremely grateful to you for pointing out this problem. We have made the latest changes in the method part.

7. Table 2 needs more detailed discussion before concluding into the listed benefit.

8. Line 257-259, what do you mean “challenging regions”. Line 265, Figure 6 should be Figure 5.

9. Line 292, Figure 5 should be Figure 6

10. Line 305 and Table2 should be consistent, model [10] or [2]?

11. The reference format should be consistent. The last few doesn’t have page no, authors’ name are also not in the right format. Please check carefully.

**The authors’ Answer:** We are extremely grateful to you for pointing out this problem. Our manuscript has been carefully revised for the above problems, for details, you can view the latest manuscript.

Thank you again for your suggestions. Your suggestions are very helpful to our research.

Reviewer #4: 1. Line 73. Achieving competitive results on the dataset is not a major contribution.

**The authors’ Answer:** We are extremely grateful to you for pointing out this problem. We have changed this content to: Our PA-Net outperformed the state-of-the-art GWC-Net [13] on the Scene Flow, KITTI 2012, and KITTI 2015 datasets.

2. Figure 1 is too far away from where is first referred. The proposed architecture is quite simple, it’s not clear what is the proposed novelty. The figure refers to the PA-Net abbreviation which can’t be found inside the figure.

**The authors’ Answer:** We are extremely grateful to you for pointing out this problem. We have modified the content of Figure 1 accordingly.

3. Please extend the argumentation in the design chose of the architecture.

**The authors’ Answer:** We are very grateful to your comments for the manuscript. We have made the latest changes in the method part.

4. Table 1 is extremely long. I could have been replaced by a better design of Figures 1, 2, 3.

**The authors’ Answer:** We are very grateful to your comments for the manuscript. After careful consideration, we deleted Table 1 because the information in Table 1 is fully introduced in the manuscript

5. May general conclusion is that the manuscript is well prepared and there are a few small ideas that are proposed. However, the overall results are just competitive, i.e. quite similar with the state-of-the-art. Therefore, I would prefer to see an extended discussion on why such a small gain is achieved. Why would someone choose the proposed method and not other state-of-the-art method. What are the main advantages of employing PA-NET?

**The authors’ Answer:** We are very grateful to your comments for the manuscript. According to your advice, we have added a discussion section in the latest manuscript.

Special thanks to you for your good comments.

Reviewer #5: The paper is very hard to follow (not unusual for a deep learning paper with so many details to the implementation). The main issue seems to be that the authors are not using the terminology in the papers they cite. The authors may have a good reason for this but it would be helpful if they either revised their paper with matching terminology or altered the reader to the difference.

**The authors’ Answer:** We are very grateful to your comments for the manuscript. We have made thorough improvements in the latest manuscript.

“concatenation cost, group-wise correlation [11], and our proposed edge 149 detection volumes (details in Section 3.3).”
What is the “concatenation cost” or group-wise correlation? The paper cited only contains a “Cost Volume”. Section 3.3 implies that the concatenation cost is equivalent to the “Cost Volume”. I am still in the dark as to what the group-wise correlation refers to.

**The authors’ Answer:** We are extremely grateful to you for pointing out this problem. We have made some improvements to introduce “the group-wise correlation and concatenation” in related works section.

As said in the introduction I could not follow the edge detection work. Why is the edge branch called an edge branch? Did you run an edge detector on the images before passing the images into the network? As written it looks like you ran two slightly different networks in parallel.

Adding an edge detection module appears to degrade the performance of the PA module (Table 2) can you comment on this?

**The authors’ Answer:** We are extremely grateful to you for pointing out this problem. In the latest manuscript, we introduced the edge detection loss and made an edge data set for end-to-end learning.

As the latest Table 1 shows, if we add the edge detection module into PSM-Net and GWC-Net, the performance will be improved greatly.

“Particular, we advocate for a parallax attention module in three dimensional(disparity, height and width) level that aims to aggregate variable disparity values. “
As I understand it your system does not in fact use the entire depth range (or a single disparity value) but two values derived from this information.

**The authors’ Answer:** We are extremely grateful to you for pointing out this problem. We have made corresponding improvements to this expression. Now the expression is “Particular, we advocate for a parallax attention module in three-dimensional (disparity, height and width) level, which structure ensures high-precision estimation by improving feature expression in near-range regions.”

“Meanwhile, finding correct correspondences in ill-posed areas (weak texture, repeat texture, and occlusion regions) remains an arduous task. “
How does your system address this problem? All your results are based on errors across an entire image not selected areas that are ill-posed.

**The authors’ Answer:** We are extremely grateful to you for pointing out this problem. We have made corresponding modification to this expression.

“To reinforce the precision of the disparity prediction in these challenging areas, in the present study, we propose a parallax attention stereo matching algorithm based on the improved group-wise correlation stereo network to learn the disparity content from a stereo correspondence. “
You don’t propose a new group-wise correlation, you use a pre-existing group-wise correlation method and extend it with an attention mechanism.
You repeatedly use the term “multi-scale” to describe your algorithm. I have always understood the term multi-scale to mean combining images of different sizes (at least in this context). What you a developing appears more “multi-featured” that “multi-scale”.

**The authors’ Answer:** We are extremely grateful to you for pointing out this problem. We have have changed “multi-scale” to “multi-featured integration”. Thank you for your advice.

Minor issues
“In recent years, researchers have attempted to combine the human attention mechanism with a 112 CNN to enhance the performance of feature extraction”
The actual human attention mechanism is still not understood, an is probably very different the systems in machine learning. Can you alter this to say something like “human-inspired”
Update citations in tables (e.g. table 2, 3)
Table 2: 1027 should be 10.27 ?

**The authors’ Answer:** We are extremely grateful to you for pointing out this problem. The latest expression is “In recent years, researchers are encouraged by the mechanism of human attention and attempt to design some network attention architectures with a CNN to enhance the performance of feature extraction.” 1027 should be 10.27, and we have changed it.

Thank you again for your sincere suggestions, which is very help to our research.

We tried our best to improve the manuscript and made some changes in the manuscript.  These changes will not influence the content and framework of the paper. We appreciate for Editors/Reviewers’ warm work earnestly, and hope that the correction will meet with approval.

Once again, thank you very much for your comments and suggestions。

Yours sincerely,

Jinan Gu
