# Supplementary material for: Parallax attention stereo matching network based on the improved group-wise correlation stereo network
Source: PLoS One. 2022 Feb 9;17(2):e0263735. doi: 10.1371/journal.pone.0263735 (PMC8827418; doi:10.1371/journal.pone.0263735)
Supplement: Attachment — Submitted filename: Response to Reviewers.docx [file pone.0263735.s003.docx]

Dear Editors and Reviewers:

Thank you for giving us the opportunity to submit a revised draft of the manuscript “Parallax attention stereo matching network based on the improved group-wise correlation stereo network” for publication in the Journal of “***PLOS ONE***”. We appreciate the time and effort that you and the reviewers dedicated to providing feedback on our manuscript and are grateful for the insightful comments and valuable improvements to our paper. We have studied comments carefully and have made correction which we hope meet with approval. Revised portion are marked in red in the paper. The main corrections in the paper and the responds to the reviewer’s comments are as flowing:

Responds to the reviewer’s comments:

Reviewer #3:

Thanks for the authors making the revision following our previous comments. Some of the problems are very well addressed. However, there are still lots of problems in the writing, not only typo or grammar, but also the logic behind the writing. I really recommend the authors to get some help. Some examples are list here:
1. The first part of the abstract should address the background, research problem and motivation. However the logic that the authors follow is that parallax information and edge contour information are not fully used. This shouldn’t be the research problem. The target is to improve the precision of disparity prediction. The author should discuss what is the current status of accuracy, how bad it is. The parallax and edge information is the methodology that the authors choose to exploit. For any research, it shouldn’t target at just experimenting some methods, that only give you the route for solving the problem not the problem itself. There are other parts in the abstract and introduction section also follow this logic which should be revised.

**The authors’ Answer:** We are very grateful to your comments for the manuscript, your suggestion is of great help to our work. According to your advice, the logical relationship is reorganized and the Abstract part was completely modified.

Firstly, the background part is “Recent stereo matching methods, especially end-to-end deep stereo matching networks, have achieved remarkable performance in the fields of autonomous driving and depth sensing.” The research problem is “However, state-of-the-art stereo algorithms, even with the deep neural network framework, still have difficulties at finding correct correspondences in near-range regions and object edge cues.” And our motivation is “To reinforce the precision of disparity prediction”.

2. In the introduction section, “However, complicated manual production steps limit their improvement.” This statement concludes too quick. It needs more details (discussion) to justify why and where these methods are not good. Probably one more sentence will be enough. Also for “and “Secondly, adopting the strategy of global attention, without increasing attention to important areas.” Why this is not good, also missing a part in the logic of the discussion.

**The authors’ Answer:** We are extremely grateful to you for pointing out this problem. Based on your advice, we have made the following improvements. Firstly, we changed “However, complicated manual production steps limit their improvement” to “However, traditional algorithms need to manually design feature description operators and cost aggregation strategies, which is not suitable for real-time applications. Complicated hand-craft production steps limit their improvement.” Secondly, we added the statement of “which is insensitive to the detailed texture regions, resulting in inaccurate disparity estimation in vital areas” to “adopting the strategy of global attention”.

3. In section 2.4, the sentence “Observation that edge detection project is conducive to improve the accuracy of disparity estimation.” is not linked clearly with the adjacent part. Also, the last sentence in the same paragraph, the author meant to criticise the Song’s work, it only mentioned multi-task instead of integration, how this become a problem? One more sentence could conclude this statement, however it is missing. For this literature review section, discussion is very important, and it should have a clear logical link, end to end, landing on the problems that this paper trying to solve.

**The authors’ Answer:** We are extremely grateful to you for pointing out this problem. We have deleted this statement of “Observation that edge detection project is conducive to improve the accuracy of disparity estimation” in the latest manuscript. Moreover, in order to express Song’s drawbacks, we added some important discussions as “However, their methods regard disparity prediction and edge detection as a multi-task learning project, those works did not establish an effective mechanism to integrate the information learned by multi-task project. As a result, the features learned by multi-task project are not effectively expressed and utilized. Focus on this problem, we construct a multi-featured integration cost volume to combine parallax features and edge features.”

4.  Section 3.3, “where matching cues are clear can be easily captured through the context pyramid.”

**The authors’ Answer:** We are very grateful to your comments for the manuscript, the new statement is “The matching clues in these regions are clear and can be easily captured through the context pyramid”.

5. Section 3.3, Figure 4 caption, “Pipeline of feature extraction network. Which includes three branches (edge detection, group-wise, and concatenation feature branches).”

**The authors’ Answer:** We are very grateful to your comments for the manuscript, we have revised the manuscript, the new title of Figure 4 is “Fig 4. Pipeline of feature extraction network.”

6. Figure 2, “Edge detection voume”, typo problem.

**The authors’ Answer:** We are extremely grateful to you for pointing out this problem. The right statement is “Edge detection volume”.

Thank you again for your suggestions. Your suggestions are very helpful to our research.

Reviewer #4: The authors answered all my comments, however, I would recommend to further polish the text and add the corresponding punctuation after the equations.

In the future, I would recommend to the authors to answer each reviewer’s comment, not just to mention that the manuscript was modified somehow, e.g.: “We have made the latest changes in the method part.”. Please add an answer to the comment and clearly present what was modified in the manuscript, not just refer to the manuscript!

**The authors’ Answer:** We are extremely grateful to you for your suggestions. In the future, we will answer each reviewer’s comment, not just to mention that the manuscript was modified.

Thank you again for your sincere suggestions, which is very help to our research.

We tried our best to improve the manuscript and made some changes in the manuscript.  These changes will not influence the content and framework of the paper. We appreciate for Editors/Reviewers’ warm work earnestly, and hope that the correction will meet with approval.

Once again, thank you very much for your comments and suggestions。

Yours sincerely,

Jinan Gu
