# Supplementary material for: Parallax attention stereo matching network based on the improved group-wise correlation stereo network
Source: PLoS One. 2022 Feb 9;17(2):e0263735. doi: 10.1371/journal.pone.0263735 (PMC8827418; doi:10.1371/journal.pone.0263735)
Supplement: Attachment — Submitted filename: Response to Reviewers.docx [file pone.0263735.s004.docx]

Dear Editors and Reviewers:

Thank you for giving us the opportunity to submit a revised draft of the manuscript “Parallax attention stereo matching network based on the improved group-wise correlation stereo network” for publication in the Journal of “***PLOS ONE***”. We appreciate the time and effort that you and the reviewers dedicated to providing feedback on our manuscript and are grateful for the insightful comments and valuable improvements to our paper. We have studied comments carefully and have made correction which we hope meet with approval. Revised portion are marked in red in the paper. The main corrections in the paper and the responds to the reviewer’s comments are as flowing:

Responds to the reviewer’s comments:

Reviewer #6

-Major concerns:
1. The only difference between the proposed method and GWC-Net is the PA module, but is it absolutely necessary? According to the qualitative results shown in the main text, it is hard to interpret why the proposed PA module is effective which only brings marginal improvements.

**The authors’ Answer:** We are very grateful to your comments for the manuscript. To prove the necessity of PA module, we add a new section (Section 4.5 Analysis and Interpretation) to exhibit PA blocks performs better than previous works. Moreover, we add a new figure map (Fig 7. Attention distribution map in KITTI 2015.) which provides an explain how the parallax attention mechanism operates in practice and why it is effective.

2. The visual comparison is not enough to support the superiority of the proposed method.

**The authors’ Answer:** We are extremely grateful to you for pointing out this problem. Based on your advice, we add a new figure (Fig 6. Training curves of PA-Net, GWC-Net and PSM-Net on KITTI 2015 dataset from epoch 100 to 300.). We can easily observation that the loss curve of PA-Net decreases more smoothly than previous works and produce consistent gains in performance which are sustained throughout the training process. Moreover, we see that PA-Net performs better than those works when the networks are trained to start fitting, and it will last achieve a highest accuracy.

3. The technical contributions of this paper are unclear. In the last few paragraphs, I think the logical relationship is not clearly explained, which leads to confusion when reading. I would like to see a description about why it works, as well as the innovations and differences from other related methods.

**The authors’ Answer:** We are extremely grateful to you for pointing out this problem. In the new manuscript, we add the works to provide an explain how the parallax attention mechanism operates in practice in Section 4.5. While PA blocks have been empirically shown to improve network performance, we would like to provide an explain how the parallax attention mechanism operates in practice. To provide a clearer picture of the behavior of PA blocks, in this section we apply several examples from GWC-Net model and examine the different distributions of sensitive respective region between 3D convolutional layers and PA blocks. We then exhibit their distribution maps in Fig 7, which is trained in the dataset of KITTI 2015.

-Minor concerns:

1. visualization of the results. All the visualizations are relatively low resolution, which is confusing and makes it hard to compare across methods and against GT.

**The authors’ Answer:** We are very grateful to your comments for the manuscript, we have revised the manuscript. in the new figure maps, all the visualizations are relatively high resolution.

2. In the paper, ‘our theory’ and ‘in this study’ appeared several times, please replace them with ‘out method’, ‘in this paper’

**The authors’ Answer:** We are extremely grateful to you for pointing out this problem. We have replaced ‘our theory’ and ‘in this study’ to ‘out method’, ‘in this paper’.

3. In the summarization of contributions, for the third contribution, revise it to: Our PA-Net achieves the accuracy of ()% on Scene flow dataset and ()% on KITTI 2015 dataset, which outperforms other methods by ()%.

**The authors’ Answer:** The new statement is “Our PA-Net achieves the accuracy of 0.775 end-point-error (EPE) on Scene flow dataset and 2.05% kitti-d1-all error on KITTI 2015 dataset, which outperforms other methods by 12%.”

4. The paper also has some minor issues in writing, in the section of Introduction
-whose goal is to -> which aims at computing
-“Learning-based stereo-matching methods through exploring feature representations and aggregation algorithms for matching costs”, this sentence is not complete.
- due to the limited learning features-> due to limited learning capability
- the most easily recognized and utilized-> the most easily recognized
-Avoid using their
-However, their methods regard….as a multi-task learning project, in this way …. effective mechanism to fuse them-> However, these methods regard disparity prediction and edge detection as a multi-task learning project. Yet, features learned in such multi-task pipelines cannot be fully exploited, which poses a great need for an effective fusion mechanism.
-In the autonomous driving task-> In the context of autonomous driving,
-Thus, it requires the disparity estimation model to provide more attention to this region.->To address this problem, more attention should be assigned to this kind of region in the disparity estimation model.
-In this study->In this paper
-we demonstrate that by designing a high-quality and efficient module for stereo matching…->we propose a high-quality and efficient module for stereo matching and our method achieves better performance on SceneFlow and KITTI than previous methods.
-It is demonstrated that based on our parallax attention stereo matching network->It is demonstrated that our parallax attention stereo matching
-edge detection task is conducive to improve the accuracy of disparity estimation? Please make the sentence more understandable.

**The authors’ Answer:** We are extremely grateful to you for your suggestions. We made the latest changes according to your suggestions in the new manuscript. Thank you very much for your careful work.

5. Section 3.2
-“However, each of the learned 3D filters with a local field that the output feature map U is unable to learn contextual information.” The whole sentence is not clear, please reorganize it. If I am understanding right. How about revising as the following: However, 3D filters learned within a local field that lacks contextual information in the output feature map U.
-when in the c-th chanel->with regard to the c-th channel
-We can treat M_u as a collector of..->The mixed feature map M_u can be treated as a collector of the local disparity texture information, and its function is to describe the entire parallax image
-“Subsequently, the descriptors” You mentioned before as “a feature map”, which is inconsistent with ‘descriptors’ used here. Please avoid confusion like this in the whole manuscript.
Also, “Subsequently” occurred twice in a paragraph. Replace the second one with ”additionally”.
-Our benefits can be summarized as follows-> our contribution can be summarized as follows\
-shorter->faster
-“increase the performance of end-point-error” increase? Is the higher value of end-point-error, the better?
-Our proposed PA module-> Our PA module
-So we can add it directly to 3D convolution layers->, which can be added directly to 3D convolution layers

**The authors’ Answer:** We are very grateful to your comments for the manuscript and we have checked through the whole manuscript Carefully. We have made corresponding modifications according to your suggestions.

6. Section 3.3
-ED features were fused-> ED features are fused
-Please elaborate the equation of group-wise cost volume after equation 6.

**The authors’ Answer:** We are extremely grateful to you for your suggestions. The new statement is “$C_{concatenation}$ and$C_{group-wise}$ are calculated as introduced in the equation of (1) and (2).”

7. Section 4.1
-It decreased 10 times->it is down-scaled by 10 when exceeding 200 epochs
-In the section of discussion, please revise “our method performance” to “our method performs...”
-Table 2 caption, “..are trained in our batch-size”->..are trained with the same batch size as our method. Please check through the whole manuscript.
-Table 3 caption, citation[10] should follow the word “test”
8. Section 4.3
- “the PA module techniques”, is PA module a technique？
- because->since
- Our proposed PA-Net performance is better-> our PA-Net performs better
- To select the proper number of->To select an optimal value of..
9. Section 4.4
- A collection of->several

**The authors’ Answer:** We are very grateful to your comments for the manuscript and we have checked through the whole manuscript Carefully. We have made corresponding modifications according to your suggestions.

Thank you again for your sincere suggestions, which is very help to our research.

We tried our best to improve the manuscript and made some changes in the manuscript.  These changes will not influence the content and framework of the paper. We appreciate for Editors/Reviewers’ warm work earnestly, and hope that the correction will meet with approval.

Once again, thank you very much for your comments and suggestions。

Yours sincerely,

Jinan Gu
